# Supplementary figures and images for: Tn5 Transposase Applied in Genomics Research
Source: Int J Mol Sci. 2020 Nov 6;21(21):8329. doi: 10.3390/ijms21218329 (PMC7664229; doi:10.3390/ijms21218329)

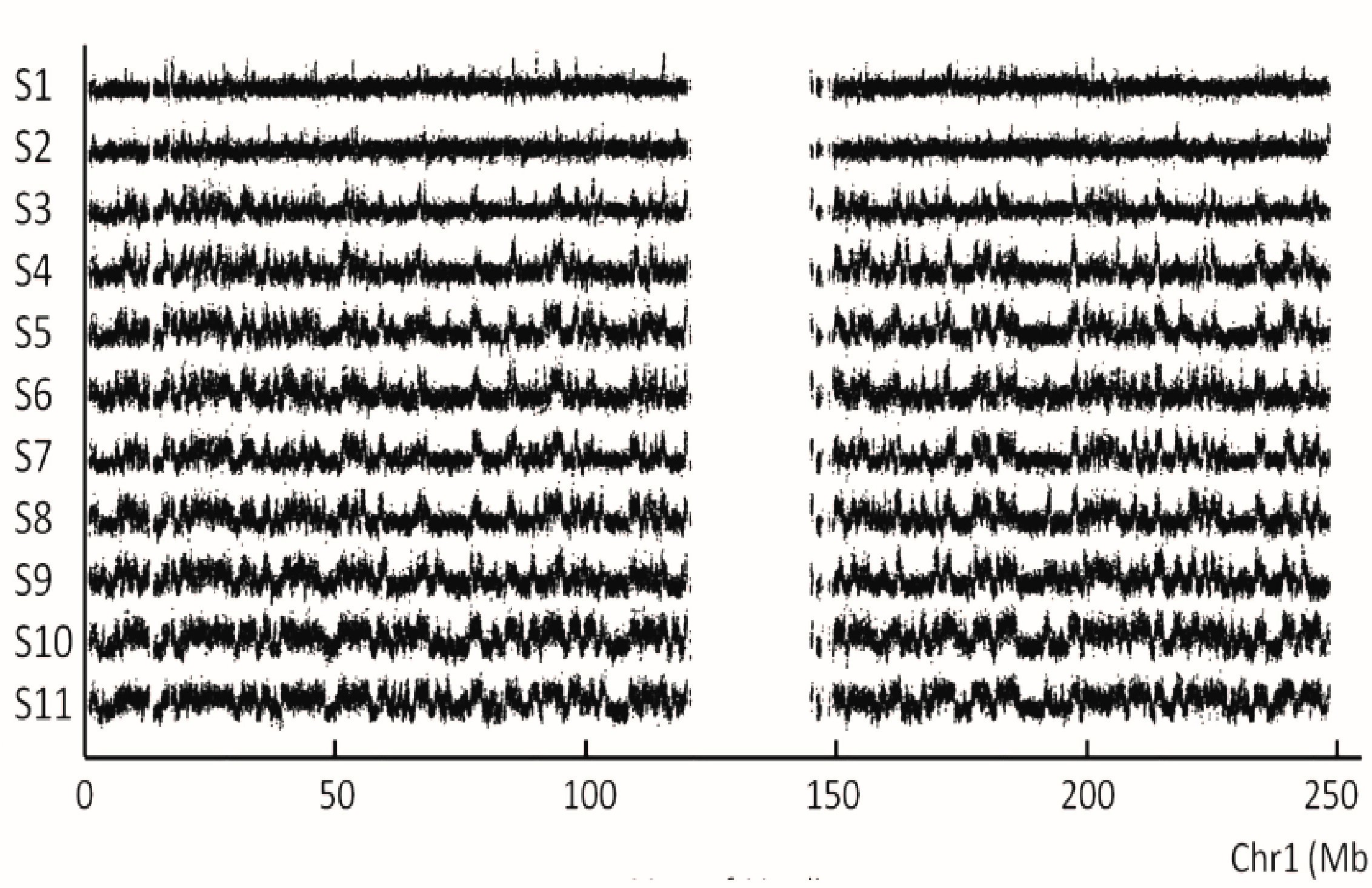

Supplement: Supplementary file 1 [file ijms-21-08329-s001.zip › supplements/supplement1.jpg]

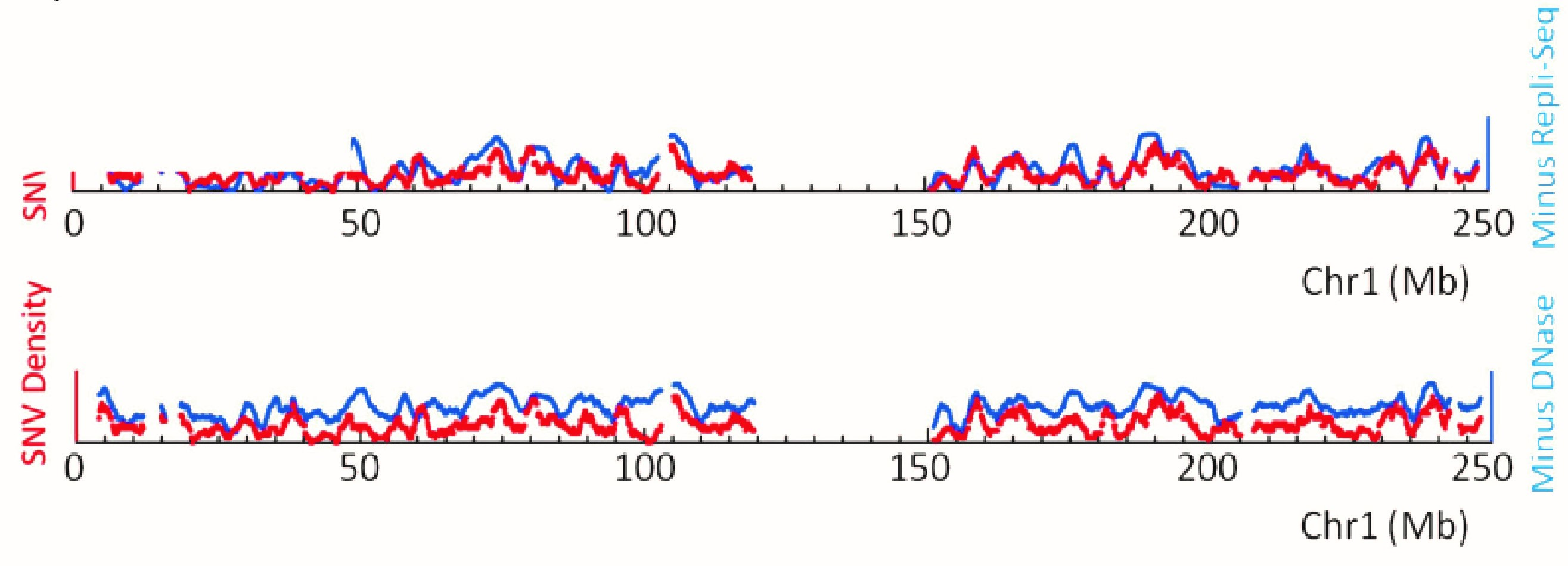

Supplement: Supplementary file 1 [file ijms-21-08329-s001.zip › supplements/supplement2.jpg]

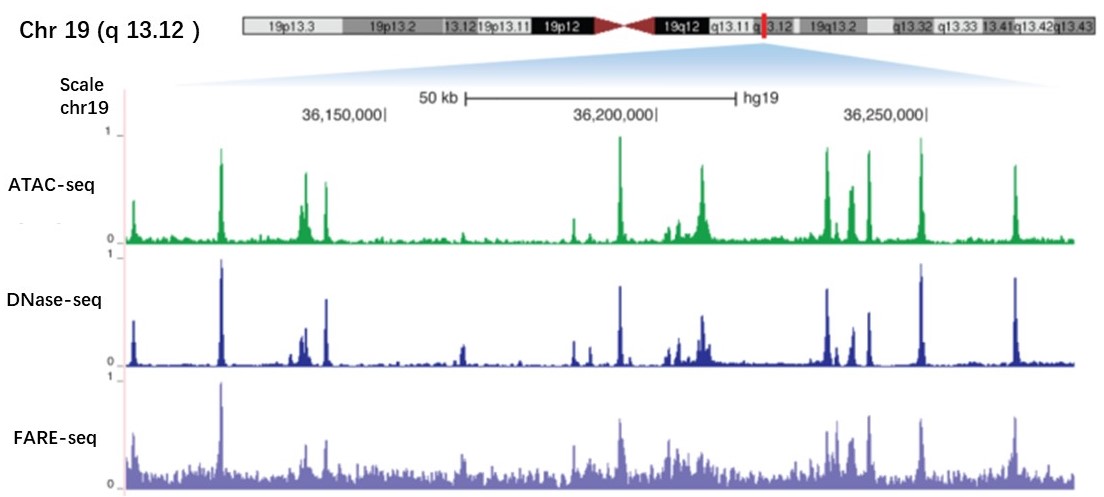

Supplement: Supplementary file 1 [file ijms-21-08329-s001.zip › supplements/supplement3.jpg]

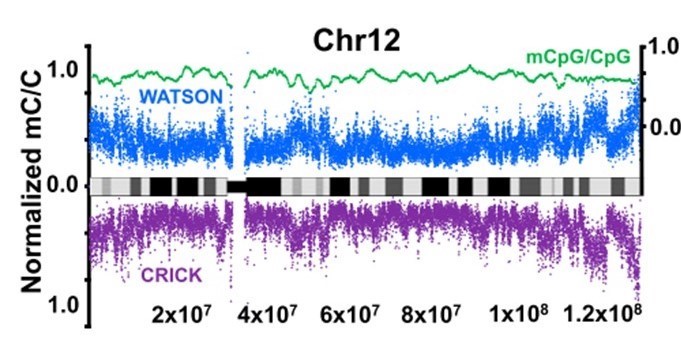

Supplement: Supplementary file 1 [file ijms-21-08329-s001.zip › supplements/supplement4.JPG]

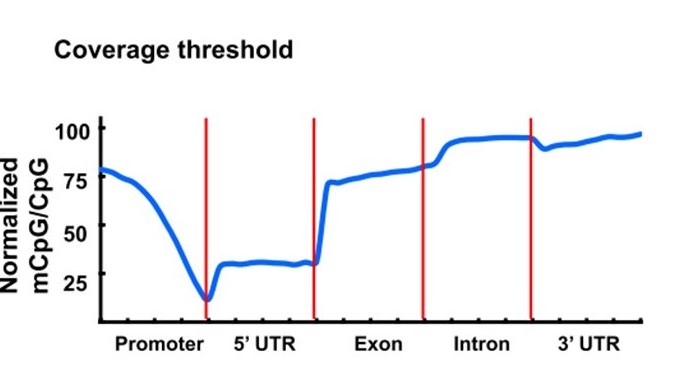

Supplement: Supplementary file 1 [file ijms-21-08329-s001.zip › supplements/supplement5.JPG]

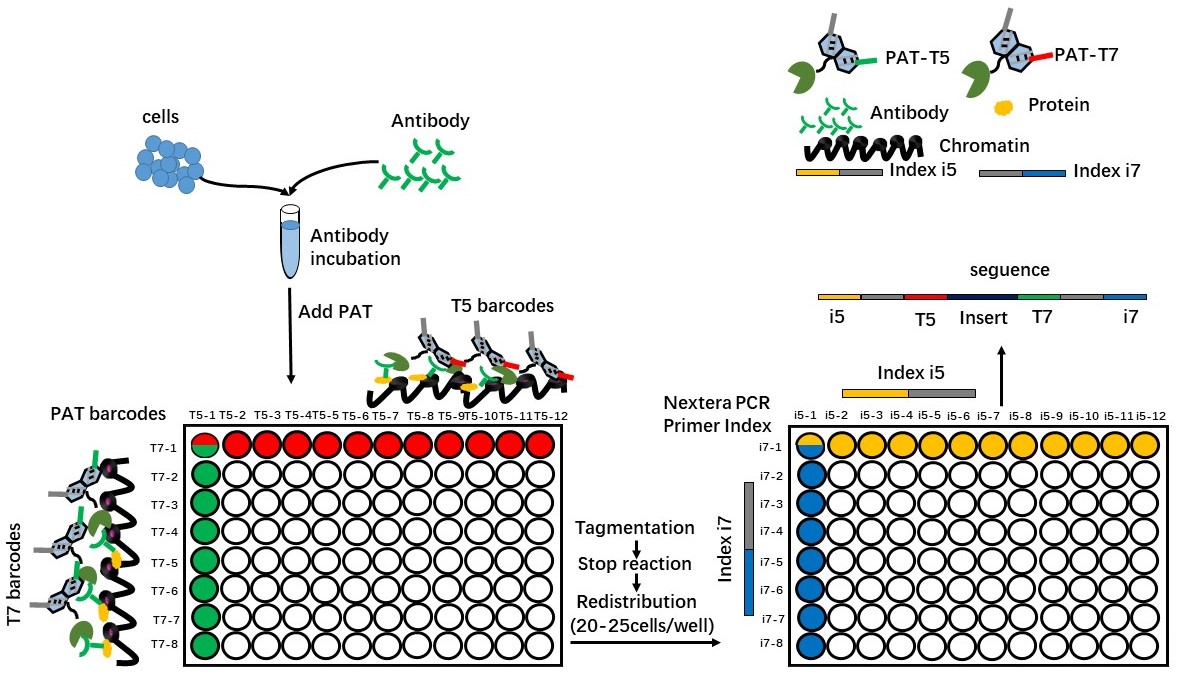

Supplement: Supplementary file 1 [file ijms-21-08329-s001.zip › supplements/supplement6.jpg]

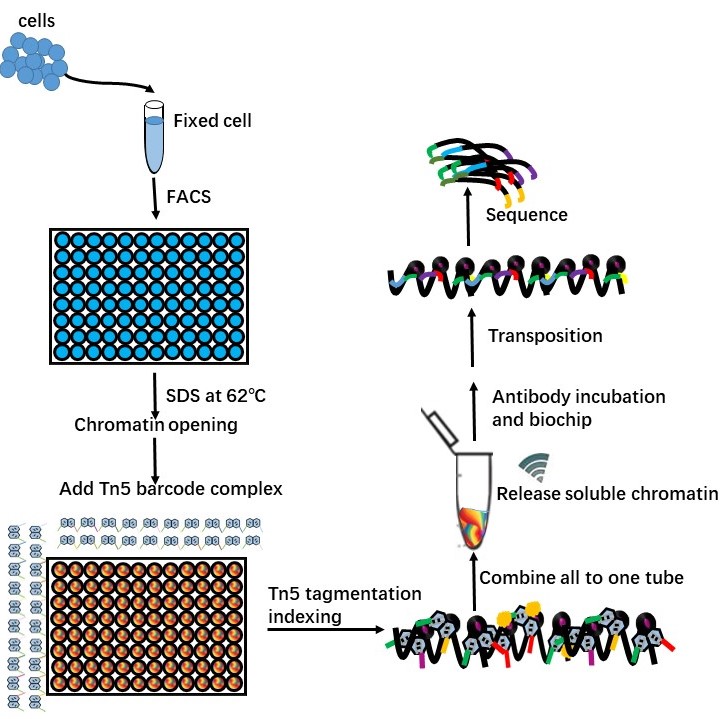

Supplement: Supplementary file 1 [file ijms-21-08329-s001.zip › supplements/supplement7.jpg]

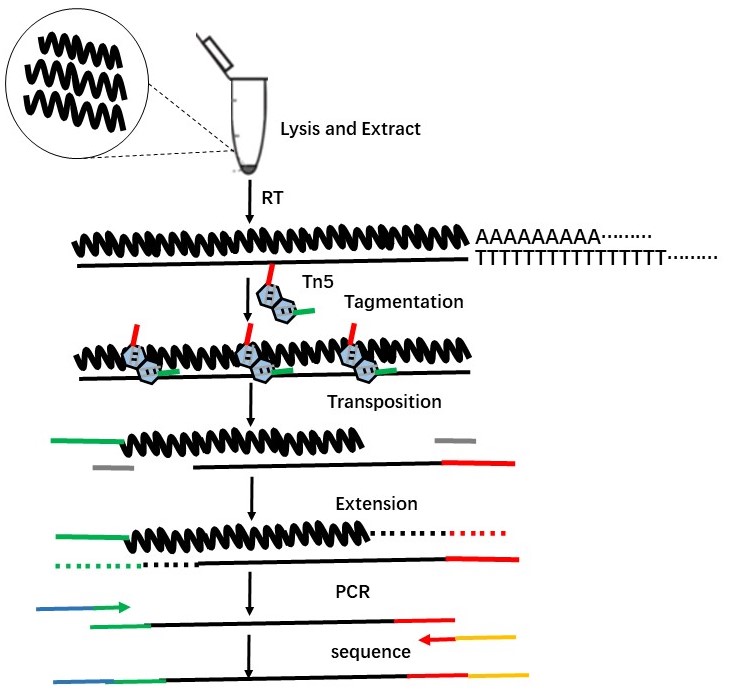

Supplement: Supplementary file 1 [file ijms-21-08329-s001.zip › supplements/supplement8.jpg]
